# Anti-Proliferative Effects of Evodiamine on Human Breast Cancer Cells

**DOI:** 10.1371/journal.pone.0067297

**Published:** 2013-06-26

**Authors:** Kai-Lee Wang, Shih-Min Hsia, Jiun-Yih Yeh, Shao-Chi Cheng, Paulus S. Wang, Shyi-Wu Wang

**Affiliations:** 1 Department of Physiology, School of Medicine, National Yang-Ming University, Taipei, Taiwan, Republic of China; 2 School of Nutrition and Health Sciences, Taipei Medical University, Taipei, Taiwan, Republic of China; 3 Department of Medical Research and Education, Taipei Veterans General Hospital, Taipei, Taiwan, Republic of China; 4 Division of Quality Assurance, Mithra Biotechnology Inc., Taipei, Taiwan, Republic of China; 5 Department of Physiology and Pharmacology, College of Medicine, Chang-Gung University, Taoyuan, Taiwan, Republic of China; 6 Ph. D. Program of Aging, College of Medicine, China Medical University, Taichung, Taiwan, Republic of China; 7 Department of Biotechnology, Asia University, Taichung, Taiwan, Republic of China; 8 The Center of General Education, National Taipei University of Nursing and Health Sciences, Taipei, Taiwan, Republic of China; University of Medicine and Dentistry of New Jersey, United States of America

## Abstract

Endocrine sensitivity, assessed by the expression of estrogen receptor (ER), has long been the predict factor to guide therapeutic decisions. Tamoxifen has been the most successful hormonal treatment in endocrine-sensitive breast cancer. However, in estrogen-insensitive cancer tamoxifen showed less effectiveness than in estrogen-sensitive cancer. It is interesting to develop new drugs against both hormone-sensitive and insensitive tumor. In this present study we examined anticancer effects of evodiamine extracted from the Chinese herb, *Evodiae fructus*, in estrogen-dependent and –independent human breast cancer cells, MCF-7 and MDA-MB-231 cells, respectively. Evodiamine inhibited the proliferation of MCF-7 and MDA-MB-231 cells in a concentration-dependent manner with concentration of 1×10^−6^ and 1×10^−5^ M. Evodiamine also induced apoptosis via up-regulation of caspase 7 activation, PARP cleavage (Bik and Bax expression). The expression of ER α and β in protein and mRNA levels was down-regulated by evodiamine according to data from immunoblotting and RT-PCR analysis. Overall, our results indicate that evodiamine mediates degradation of ER and induces caspase-dependent pathway leading to inhibit proliferation of breast cancer cell lines. It suggests that evodiamine may in part mediate through ER-inhibitory pathway to inhibit breast cancer cell proliferation.

## Introduction

The discovery and subsequent development of novel chemical entities into anti-cancer drugs are one of the major goals of oncologists. Compounds from traditional Chinese medicine have been examined for their anti-cancer potential in recent decades, and for those compounds that do exhibit such potential, the delineation of their mechanism of action may have an enormous influence on the development of new strategies in cancer therapies. Evodiamine is one of the important components of Chinese herb Wu-Chu-Yu. Numerous reports have revealed the effects of evodiamine including anti-angiogenesis [1], anti-tumor growth [2], [3], anti-invasive and metastatic activities [4], [5], [6], up-regulating apoptosis [6] anti-allergic effects [7], and anti-inflammatory effects [8]. Some of these results demonstrate that evodiamine exhibits inhibitory effects on the growth and metastasis of cancer cells both *in vitro* and *in vivo*.

Breast cancers can be categorized into estrogen sensitive and estrogen insensitive patterns according to the expression of estrogen receptor (ER). Many drugs have been developed like ER antagonists (tamoxifen and clomiphene) [9], or aromatase inhibitors [10] to diminish the proliferative effects of estrogen. However, the mechanisms of how evodiamine affect the hormone-dependent cancer cells like breast cancer are poorly defined. In this study, we investigated the effects of evodiamine on the proliferation of both estrogen-dependent and independent breast cancer cell lines MDA-MB-231 and MCF-7. The intracellular signaling pathway of apoptosis and the expression of ERs were also investigated.

## Materials and Methods

### 2.1 Compound

Evodiamine (EVO) was provided by Dr. L.C. Lin, National Research Institute of Chinese Medicine, Taipei, Taiwan, ROC. The chemical structure of EVO has been shown [2]. EVO was dissolved in dimethylsulfoxide (DMSO). The stock was stored at −20°C and then diluted in medium before each experiment. The final DMSO concentration did not exceed 0.1% throughout the study.

### 2.2 Culture with or without Evodiamine of Breast Cancer Cell Lines

The human breast cancer cell lines, including MCF-7 and MDA-MB-231 were purchased from Bioresource Collection and Research Center, Taiwan, R. O. C. MCF-7 is estrogen receptor-alpha-positive human breast cancer cell line and MDA-MB-231 is highly-metastatic and estrogen receptor-negative human breast cancer cell line. MCF-7 and MDA-MB-231 were cultured in minimum essential medium (MEM, Gibco Laboratories, Buffalo, Grand Island, NY, USA) supplemented with 10% of fetal bovine serum (FBS, Biological Industries, KBH, Israel), 50 IU/ml of penicillin G (Sigma, St. Louis, MO, USA), and 50 µg/ml of streptomycin (Sigma). The MEM for MCF-7 cell line was supplemented with 0.01 mg/ml of bovine insulin (Sigma) as previously described [11]. Cells were cultured at 37°C under aeration with 95% air +5% CO_2_ and incubated with or without evodiamine (1×10^−7^∼1×10^−5 ^M).

### 2.3 Cell Proliferation-MTT Assay

The colorimetric [3-(4,5-dimethylthiazol-2-yle)2,5-diphenyltetrazolium bromide] (MTT) assay was modified and performed to quantify the cell proliferation. Briefly, in the continuous treatment procedure, cells were incubated in 96-well microplates (Falcon, Franklin Lakes, NJ, USA) with MEM supplemented with 10% FBS. After 48 hr, the media was removed and replaced by either medium containing different concentration of drug or a drug-free medium (control condition). After 24, 48, 72 and 96 hrs, the media were removed and replaced by 50 µl of 1 mg/ml MTT (Sigma) in RPMI 1640 medium. After incubation for 6 hr, the MTT solution was removed and replaced by 100 µl of DMSO, and the plates were shaken for 3 min. The optical density of each condition was determined using a microplate reader (Dynatech Laboratories, Chantilly, VA, USA) at a wavelength of 570 nm with a reference wavelength of 630 nm. Each experimental condition was repeated 3 times.

### 2.4 Western Blot

The method of Western blotting has been described elsewhere [12], [13]. Cells after treatment were harvested and washed twice with cold PBS. The cell pellets were lysed in RIPA lysis buffer [50 mM Tri-HCl (pH 7.4), 1% NP-40, 0.25% Na-deoxycholate, 150 mM NaCl, 1 mM EDTA, l mM phenylmethylsulfonyl fluoride, 1 µg/ml aprotinin, 1 µg/ml leupeptin, 1 µg/ml pepstatin, 1 mM Na-orthovanadate and 1 mM NaF]. Cell lysates were homogenized using syringe with 26 gauge needle and insoluble material was pelleted by centrifugation at 14,000 *g* at 4°C for 15 min. The protein concentration in the cell lysate was determined by Bradford assay [14]. Equal amounts of protein were size-fractionated by SDS-PAGE and electro-transferred to nitrocellulose membranes (Schleicher & Schuell, Inc., Keene, NH, USA). The membranes were blocked with 5% nonfat milk in TBST solution [20 mM Tris-HCl (pH 7.6), 135 mM NaCl and 0.1% Tween 20]. Caspase-7 was detected by rabbit anti-human antibody at a dilution of 1∶1000 (Cell Signaling Technology Inc., Danvers, MA, USA). Poly (ADP-ribose) polymerase (PARP) was detected by mouse monoclonal antibody at a dilution of 1∶6000 (Cell Signaling Technology Inc.). Bax was detected by mouse monoclonal antibody at a dilution of 1∶250 (BD Transduction Laboratories, Becton Dickinson, San Diego, CA. USA). Estrogen receptor alpha (ERα) was detected by mouse monoclonal antibody at a dilution of 1∶1000 (Cell Signaling Technology Inc.). Estrogen receptor beta (ERβ) was detected by rabbit antibody at a dilution of 1∶4000 (Upstate Biotechnology Inc. Lake Placid, NY, USA). Immunodetection of Nbk (Bik) was performed using a polyclonal rabbit anti-Nbk antiserum at a dilution of 1∶1000 (Cell Signaling Technology Inc.), followed by biotinylated anti-rabbit IgG antiserum and horseradish peroxidase-conjugated streptavidin [15].

### 2.5 Reverse Transcription Polymerase Chain Reaction (RT-PCR)

Total cellular RNA of fresh isolated cells was isolated using TRIzol Reagent (Invitrogen, Carlsbad, CA, USA). cDNA was synthesized using M-MuLV reverse transcriptase (New England Biolabs, Ipswich, MA, USA). Specific gene product was amplified by PCR reaction with Taq DNA polymerase (Invitrogen). Primer sets for PCR were listed as following:

ERα sense strand:5′ -GTTCCCTACCGCCTCCACTC-3′;

ERα antisense strand, 5′-TACCAAGAGAAGCCCGAGCAG-3′;

Nbk/Bik sense strand : 5′ –GCCAGAGGAGAAATGTCTGA-3′;

Nbk/Bik antisense strand : 5′ -AGTGTGGTGAAACCGTCCAT-3′;

GAPDH sense strand : 5′–CCACCCATGGCAAATTCCATGGCA-3′;

GAPDH antisense strand : 5′ -TCTAGACGGCAGGTCAGGTCCACC-3′.

### 2.6 Statistical Analysis

For all groups data are presented as the mean plus or minus standard error of the mean (SEM). Statistical analysis was performed by one-way analysis of variance (ANOVA) followed by Duncan’s multiple range test of the difference in group means compared with control mean, using the SPSS [16]. The difference between two means was considered statistically significant when *p*<0.05.

## Results

### 3.1 Inhibitory Effects of Evodiamine on the Growth of Estrogen-sensitive, -insensitive Breast Cancer Cells

**Figure 1:**
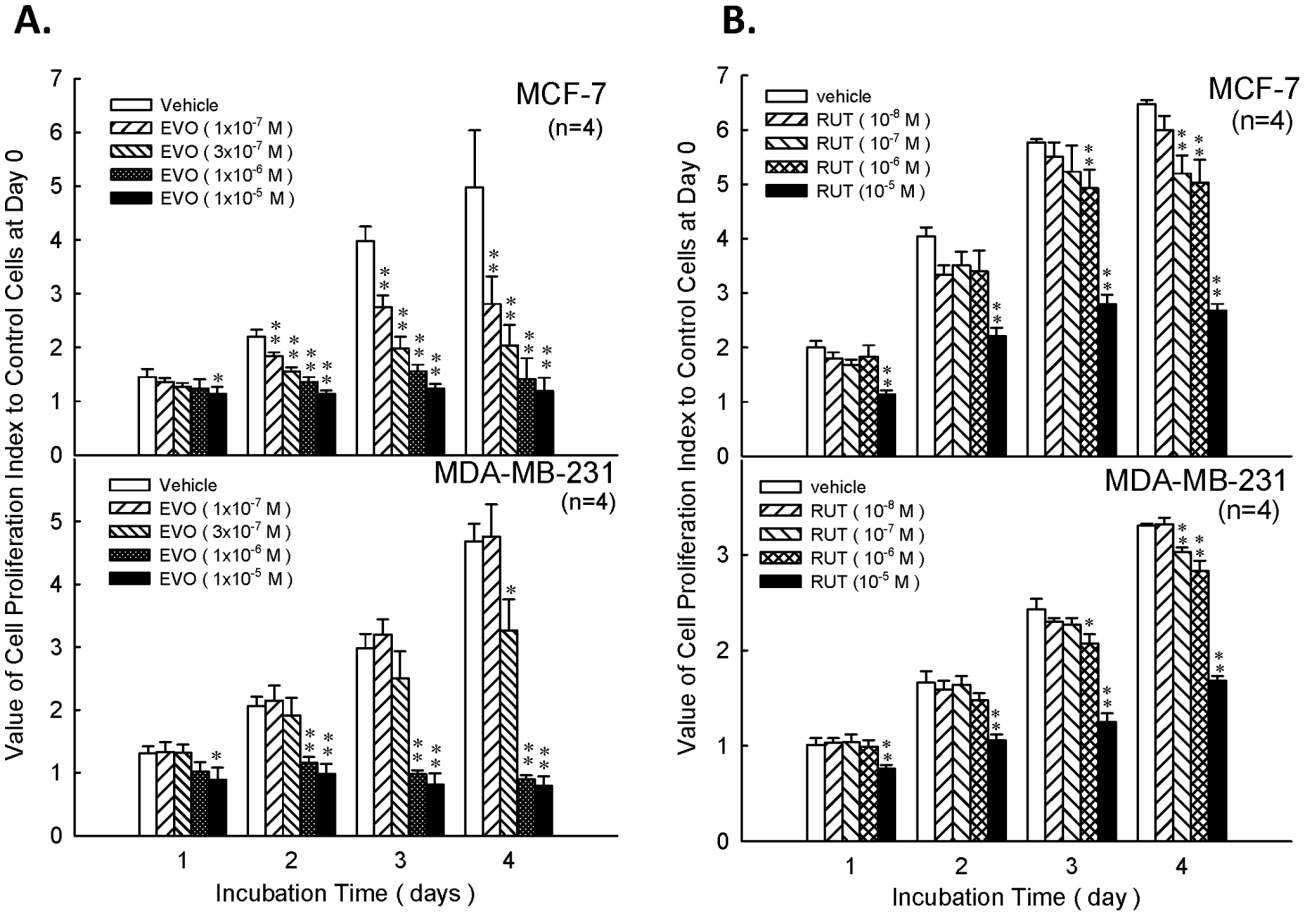
Effects of evodiamine(EVO)on the proliferation of MCF-7 and MDA-MB-231. The incubation period was from 1 to 4 days. Proliferation index was measured by MTT assay. Each value presents mean plus or minus SEM. * *p*<0.05, ** *p*<0.01 as compared to corresponding vehicle group.

**Figure 2:**
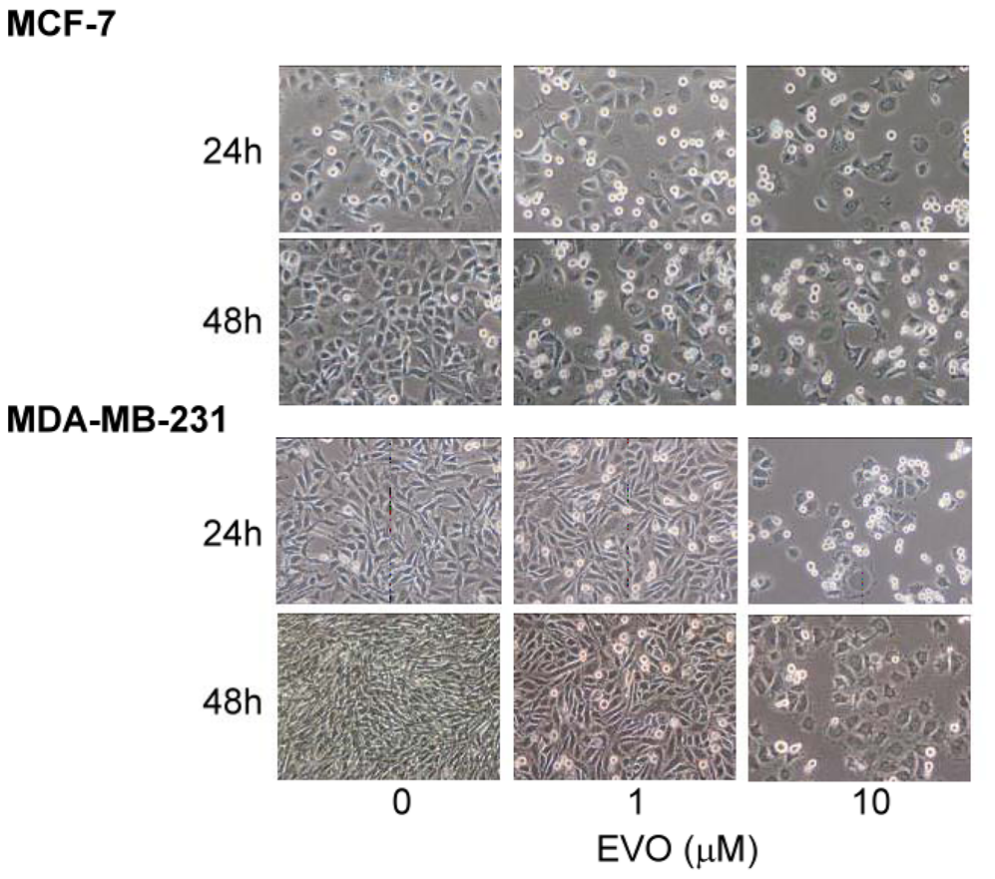
Morphological change of MCF-7 and MDA-MB-231 cells after administration with evodiamine (1×10 ^−6^, 1×10^−5^ M) for 24 or 48 hrs.

The morphological changes of MCF-7 and MDA-MB-231 cells were shown in **Figure 1**. As shown in **Figure 2**, evodiamine exhibited dose-dependently inhibitory effects on the proliferation of estrogen-dependent cells MCF-7 and estrogen-independent cells MDA-MB-231. On day 2 of incubation, the lowest concentration (1×10^−7^ M) of evodiamine expressed the inhibitory effect which was similar to the effects caused by higher concentrations (3×10^−7^, 1×10^−6^ and 1×10^−5^ M). The similar inhibitory types were also observed on day 3 and day 4. However, the inhibitory effect was lower in MDA-MB-231 cells as compared with MCF-7 cells.

### 3.2 Effects of Evodiamine on the Expression of Procaspase 7 and Caspase 7 in MCF-7 Cells

**Figure 3:**
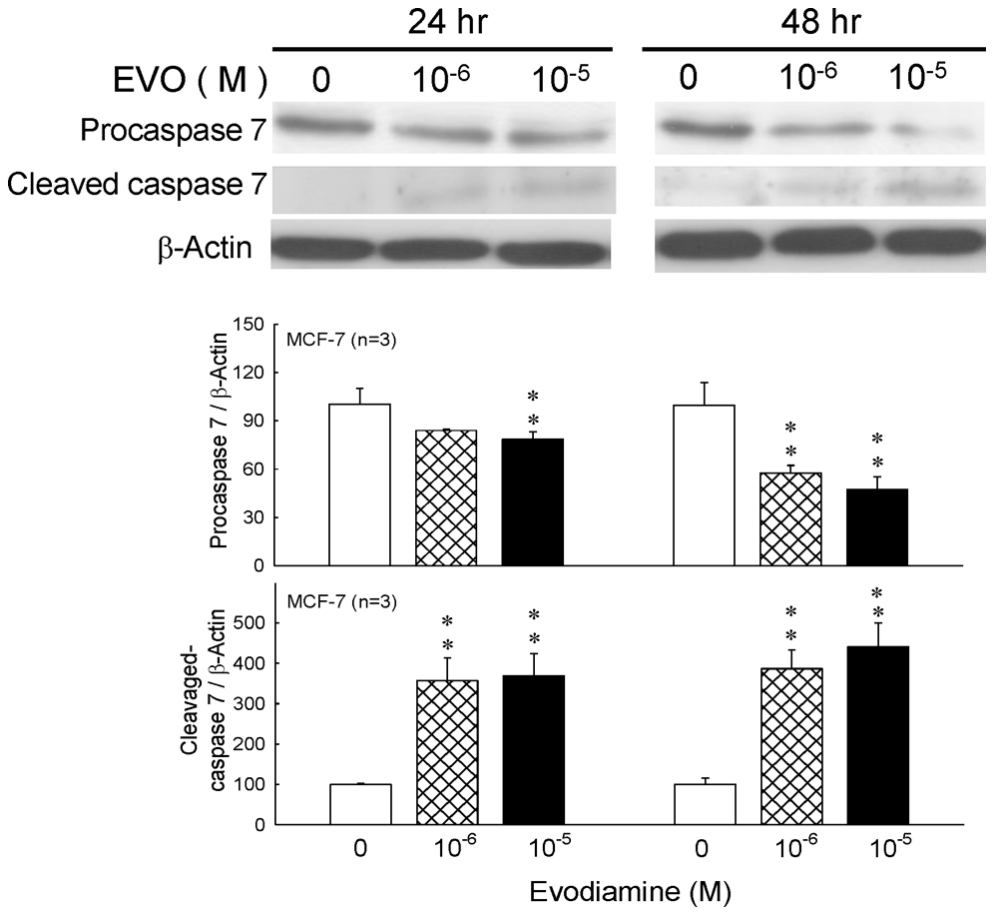
Effects of evodiamine(EVO)on the protein expression of procaspase 7 and cleaved-caspase 7 in MCF-7 cells treated with evodiamine for 24 or 48 hr. Cell lysates were analyzed by Western blot. Each value presents mean plus or minus SEM. ** *p*<0.01 as compared to corresponding vehicle group.

After treatment of evodiamine at 0, 1×10^−6^, or 1×10^−5^ M for 24 or 48 hrs, MCF-7 cells were lysed for detection of the expressions of procaspase 7 and cleaved caspase 7 by Western blot. **Figure 3** indicats that the expression of procaspase 7 was decreased after 24 and 48 hrs of evodiamine treatment. Oppositely, the expression of cleaved caspase 7 was increased significantly.

### 3.3 Effects of Evodiamine on the Expression of PARP and Cleaved PARP in MCF-7 Cells

**Figure 4:**
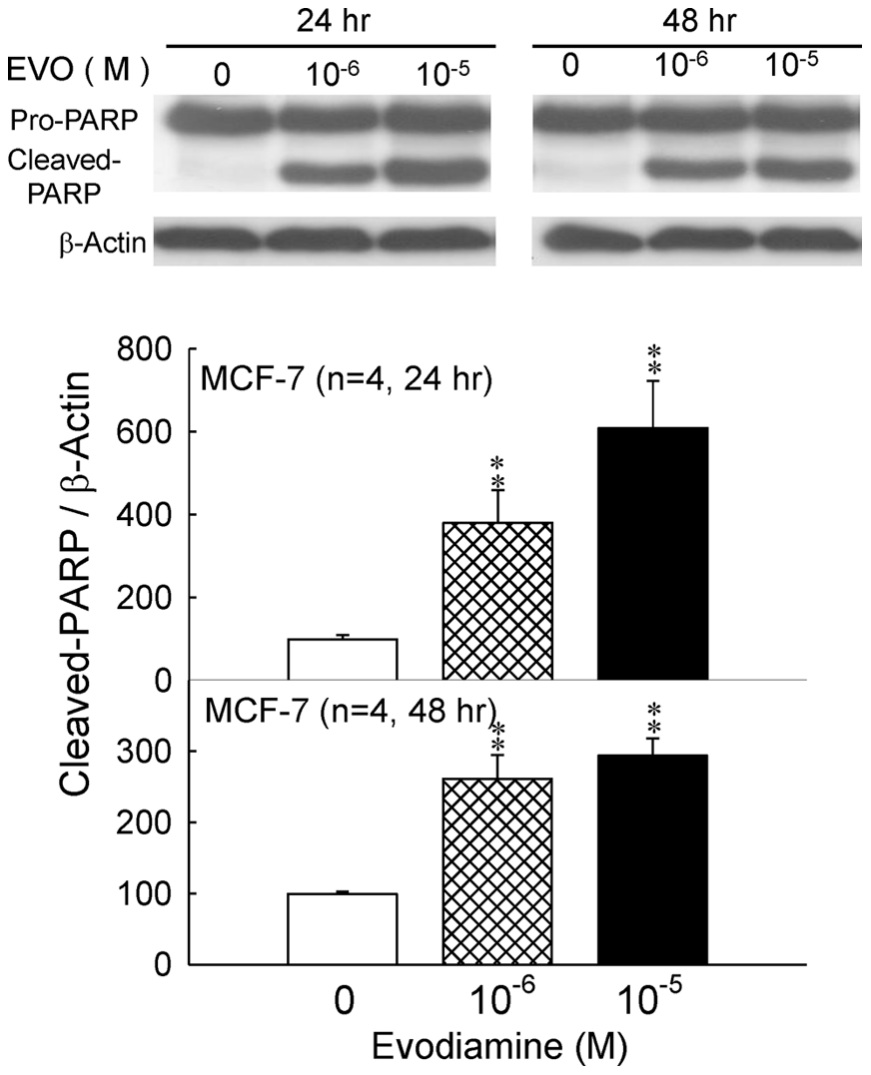
Effects of evodiamine (EVO) on the protein expression of PARP and cleaved-PARP in MCF-7 cells treated with evodiamine for 24 or 48 hr. Cell lysates were analyzed by Western blot. Each value presents mean plus or minus SEM. ** *p*<0.01 as compared to corresponding vehicle group.

PARP is a 116 KDa protein involved in the DNA repair, differentiation and chromatin structure formation. PARP is cleaved by caspase 3 during apoptosis, and possibly other caspases, into an 89 KDa fragment [17]. After being treated with evodiamine for 24 or 48 hrs at the concentration of 1×10^−6^ or 1×10^−5^ M, the nuclear proteins of MCF-7 cells were extracted, and then the PARP and cleaved PARP were analyzed by Western blot. Beta-actin was adopted as an internal control protein. **Figure 4** indicated that the expression of cleaved PARP increased significantly after being treated with evodiamine at the dosages and time periods described above (*p*<0.01).

### 3.4 Effect of Evodiamine and Anti-estrogen Agent (ICI-182,780) on the Proliferation of MCF-7 Cells

**Figure 5:**
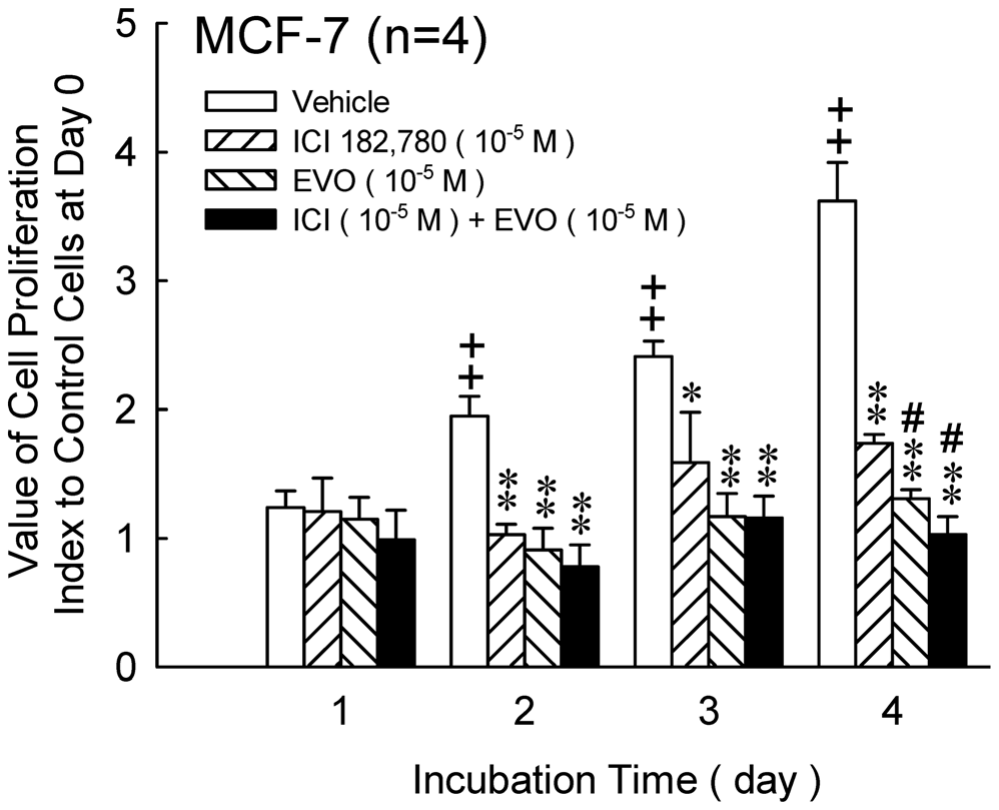
Effects of evodiamine(EVO)on the mRNA (a.) and protein (b.) expression of Nbk (Bik) in MCF-7 cells which were treated for 18 hrs. The media for MCF-7 were phenol red-free DMEM/F12 supplemented with 2% charcoal/dextran-stripped FBS and estradiol(10^−9^ M). Cell lysates were analyzed by RT-PCR and Western blot. Each value presents mean plus or minus SEM. ** *p*<0.01 compared to vehicle group.

To compare the effects of evodiamine with ICI-182,780, MCF-7 cells were treated with evodiamine (1×10^−5^ M), ICI-182,780 (1×10^−5^ M), or the combination of the two agents for 1 to 4 days. The cell proliferation was detected by MTT assay. As indicated in **Figure 5**, from day 2 to day 4, proliferations of MCF-7 cells were significantly inhibited by ICI-182,780, evodiamine, or the combination of the two agents. Only at 4-day treatment, the group of evodiamine only and evodiamine combined with ICI-182,780 displayed a significantly lower proliferation index compared with group of ICI-182,780. The group of ICI-182,780 combined with evodiamine displayed a synergistic effect.

### 3.5 Effects of Evodiamine on the Expression of ERα and ERβ in MCF-7 Cells

**Figure 6:**
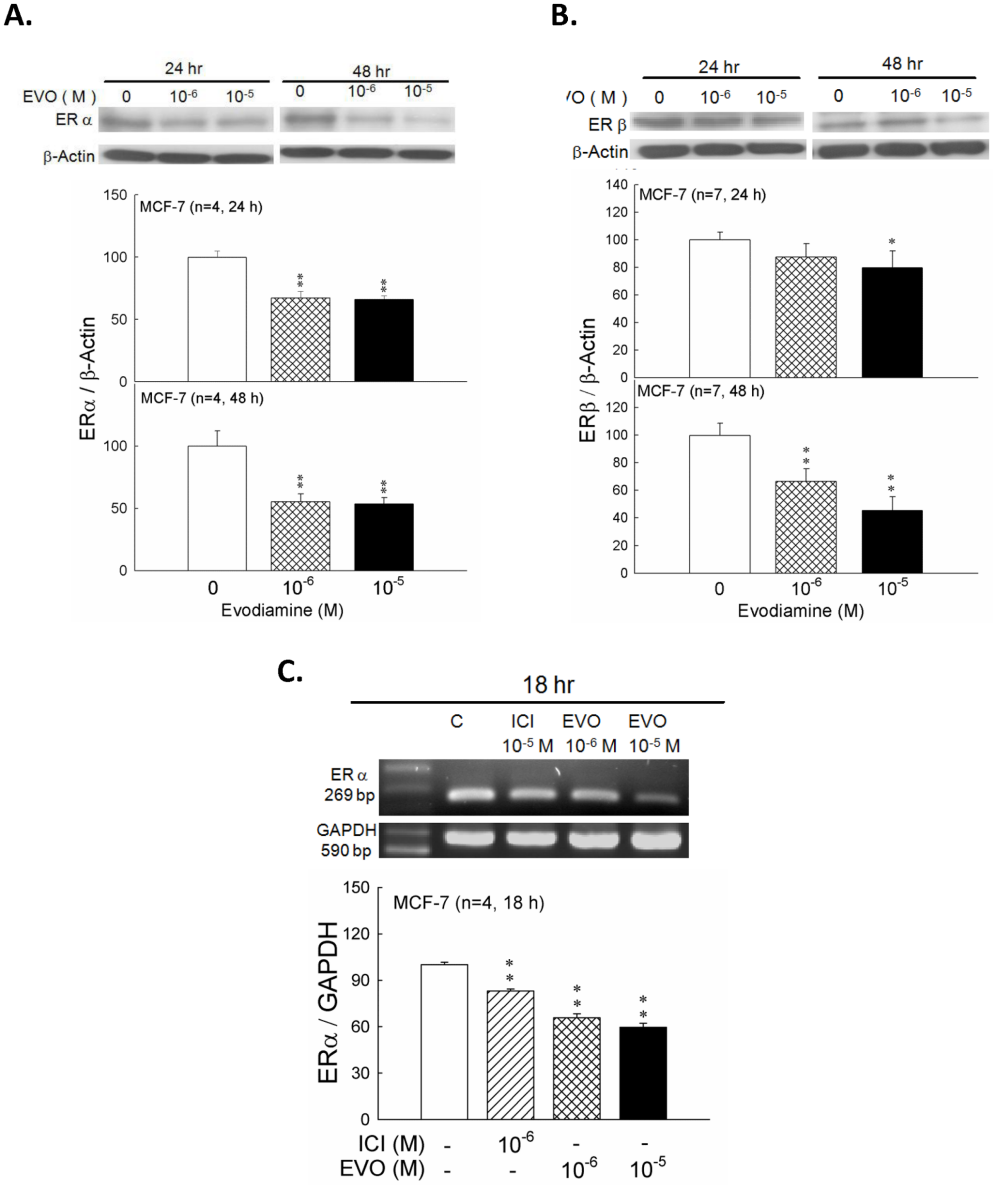
Effects of evodiamine(EVO)on the protein expression of Bax in MCF-7 cells which were treated for 18 hrs. The media for MCF-7 cells were phenol red-free DMEM/F12 supplemented with 2% charcoal/dextran-stripped FBS and estradiol(10^−9^ M). Cell lysates were analyzed by Western blot. Each value presents mean plus or minus SEM. ** *p*<0.01 as compared to vehicle group.

After being treated with evodiamine at 1×10^−6^ or 1×10^−5^ M for 24 or 48 hrs, the intracellular proteins were extracted for estrogen receptor detection. The expressions of ERα were significantly inhibited by evodiamine at 1×10^−6^ and 1×10^−5^ M for 24- and 48-hr treatments. After evodiamine treatment, evodiamine significantly inhibited ERα expression (*p*<0.01). Evodiamine at 1×10^−5 ^M inhibited the expression of ERα markedly (*p*<0.01) (**Figure 6** **,** **a** and **b**).

According to the result of protein expression described above, ERα was more sensitive to evodiamine than ERβ. We focused on the mRNA expression of ERα in the subsequent study. MCF-7 cells were treated with evodiamine at 1×10^−6^ and 1×10^−5^ M and ICI-182,780 at 1×10^−5^ M for 18 hrs. Cells were then harvested for the detection of mRNA of ERα. Results revealed that ICI-182,780 at 1×10^−5^ M, evodiamine at 1×10^−6^ M and 1×10^−5^ M attenuated the expression of ERα mRNA (**Figure 6c**). These mRNA changes were correspondent with the results of protein expression after being treated with evodiamine.

### 3.6 Effect of Evodiamine on the Protein Expression of Bax and Nbk in MCF-7 Cells

**Figure 7:**
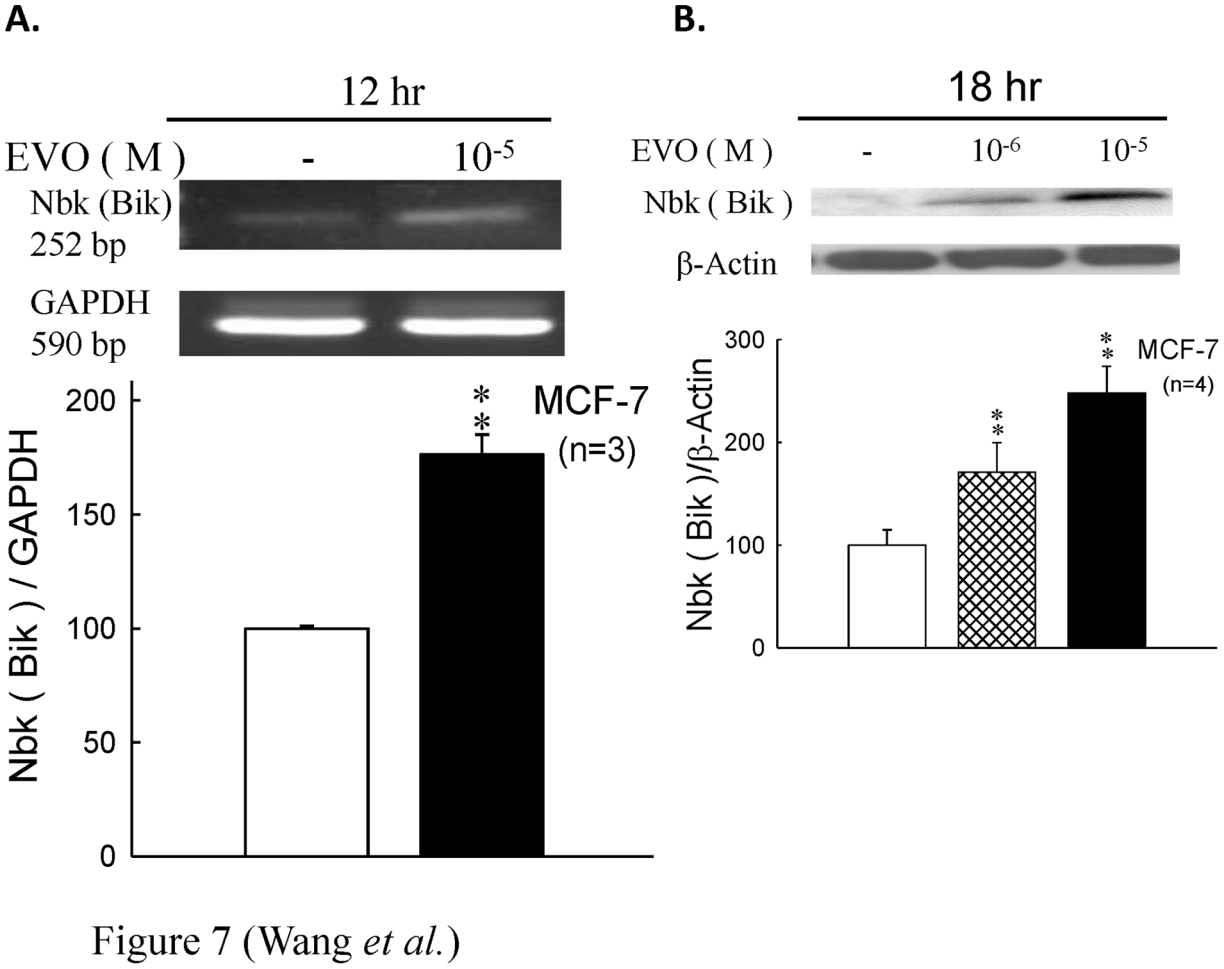
Effects of ICI-182,780(ICI), evodiamine(EVO)and ICI combined with EVO on the proliferation of MCF-7 cells. The incubation period was from 1 to 4 days. Proliferation index was measured by MTT assay. Each value presents mean plus or minus SEM. ++*p<*0.01 as compared to vehicle of day 1, *p<0.05 and **p<0.01 as compared to corresponding vehicle group. #*p<*0.05 as compared to ICI treated group.

Furthermore, the expression of pro-apoptotic bcl-2 family member, Bax and Nbk, were also examined (**Figure 7** **and** **8**). MCF-7 cells treated with evodiamine for 12 and 18 hrs could significantly up-regulate the expression of Nbk and Bax.

**Figure 8:**
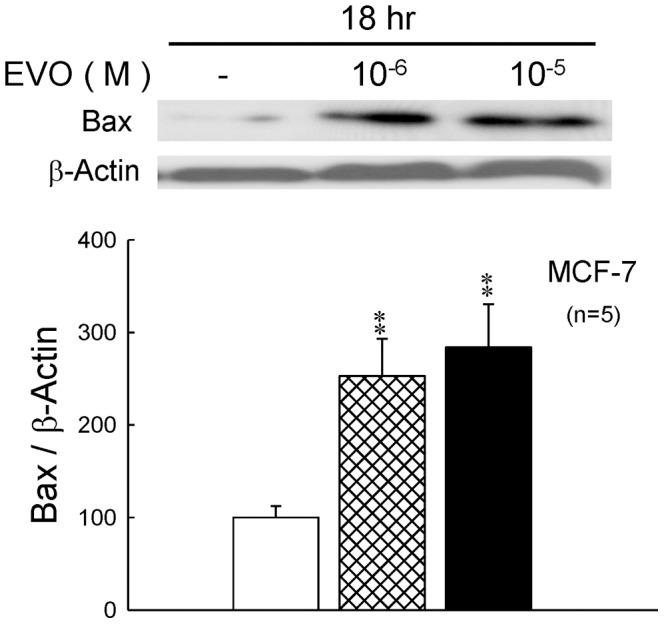
Effects of evodiamine (EVO) on the protein expression of ERα (a.), ERβ (b.) and mRNA of ERα (c.) in MCF-7 cells which were treated with evodiamine for 24 or 48 hr for the detection of protein, and 18-hrs culture was for mRNA measurement. Cell lysates were analyzed by Western blot (**a. and b.**) or RT-PCR (**c.**). Each value presents mean plus or minus SEM. **p<0.01 as compared to corresponding vehicle group.

## Discussion

In this study we found the evodiamine significantly inhibited the proliferation of both estrogen-dependent and estrogen-independent cell lines MCF-7 and MDA-MB-231, respectively. However, the MCF-7 cells were more sensitive to evodiamine than MDA-MB-231 cells in lower doses and shorter reaction time period. According to these results, we suggested that the mechanisms of proliferation-inhibitory effects of evodiamine on these two kinds of cell lines are different. Meanwhile, in our previous study, the lactate dehydrogenase (LDH) assay indicated that MCF-7 cells treated by evodiamine at 10^−7^, 10^−6^, or 10^−5^ M did not cause different cytotoxicity level (data not shown). This result indicated that the decreased proliferation effect of evodiamine on MCF-7 cells was through apoptosis rather than necrosis approach. The apoptosis-related mechanism and the niche of estrogen receptors were then investigated. Caspases can be categorized into initiator caspases (caspase 8, 9, 10) and effector caspases (caspase 3, 7). The major effect of caspase is protein hydrolysis and it leads to the change of cell morphology and DNA fragmentation [18]. Caspase can also digest PARP into cleaved-PARP and lead to DNA fragmentation.

According to previous studies, MCF-7 cell did not express caspase 3 [19], [20], we chose caspase 7 and PARP as the indicators of apoptosis. PARP is a protein involved in a number of cellular processes involving mainly DNA repair and programmed cell death. Full-length PARP is an 116 kDa protein involved in the repair of DNA, in differentiation and in chromatin structure formation. During apoptosis, PARP is cleaved by caspase-3, and possibly other caspases, into an 89 kDa fragment [17]. It has been shown that evodiamine treatment increases the expression of cleaved caspase 7 and PARP in the present study, and the results reveal that inhibitory effects of evodiamine on proliferation of MCF-7 cells are through apoptosis rather than necrosis indeed (**Figures 1** **,** **2** **,** **3** **,** **4**).

To investigate the anti-estrogen effects of evodiamine, we performed a test according to the theory proposed by Nolan [21], that is, if two compounds affect some cells through same mechanism, the effects induced by individual treatment of one of the drugs will not differ from the treatment of combination of the two compounds. Hence, an pure anti-estrogen compound ICI-180,782 was selected. A derivative of estradiol with a long, hydrophobic side chain at the 7 alpha position, ICI-182,780 demonstrates a pure antiestrogenic profile on all genes and in all tissues studied to date [22]. The mechanism of action of this steroidal antiestrogen differs significantly from other SERMs with mixed agonist/antagonist properties. In contrast to other SERMs, ICI-182,780 blocks ER transactivation coming from both AF-1 and AF-2 domains [23]. The drug may also impair ER dimerization, but most importantly, ICI-182,780 induces ER degradation, with a marked reduction in the cellular concentration of ER [24], [25], [26]. In the present study, ICI-182,780 (10^−5 ^M), evodiamine (10^−5^ M) and ICI-182,780 (10^−5^ M) plus evodiamine (10^−5^ M) were treated to MCF-7 cells for 24 to 96 hrs. As indicated in **Figure 5**, no significant difference was observed at 24, 48, and 72 hrs following treatments. These results imply that the inhibitory effects of evodiamine on cell proliferation are similar to ICI-182,780 which is demonstrated through antiestrogenic and ER degradation pathway.

In our current study, we had also found that ER protein expression as well as mRNA levels were decrease after evodiamine treatment (**Figure 6**). This phenomenon is similar to ICI-182,780 treatment [24], [25], [26]. Moreover, previous study has found that the expression of ERα plays an important role in IGF-1 signaling pathway [27], down-regulation of ERα by chemicals or specific siRNA reduces cell proliferation index [28], [29]. Therefore, we suggest that evodiamine could inhibit breast cancer cell proliferation through ER-inhibitory pathway. However, the mechanisms of ER degradation and cell apoptosis are still unclear and the accurate mechanism of evodiamine has needed further investigated.

The Bcl-2–associated X protein, or BAX gene was the first identified pro-apoptotic member of the Bcl-2 protein family [30]. Bax is a pro-apoptotic Bcl-2 protein containing BH1, BH2 and BH3 domains. In healthy mammalian cells, the majority of Bax is found in the cytosol, but upon initiation of apoptotic signaling, Bax undergoes a conformation shift, and inserts into organelle membranes, primarily the outer mitochondrial membrane [31]. Bax is believed to interact with, and induce the opening of the mitochondrial voltage-dependent anion channel (VDAC). Alternatively, growing evidence suggests that activated Bax and/or Bak form an oligomeric pore, MAC in the outer membrane. This results in the release of cytochrome c and other pro-apoptotic factors from the mitochondria, often referred to as mitochondrial outer membrane permeabilization, leading to activation of caspases. This defines a direct role for Bax in mitochondrial outer membrane permeabilization, a role common to the Bcl-2 proteins containing the BH1, BH2 and BH3 domains. BCL2-interacting killer (apoptosis-inducing), also known as BIK, is a protein known to interact with cellular and viral survival-promoting proteins, such as BCL2 and the Epstein-Barr virus in order to enhance programmed cell death. Because its activity is suppressed in the presence of survival-promoting proteins, this protein is suggested as a likely target for antiapoptotic proteins. This protein shares a critical BH3 domain with other death-promoting proteins, Bax and Bak [32]. Bik is induced in MCF-7/BUS cells in the absence of estrogen signaling and plays a critical role in the antiestrogen-provoked breast cancer cell apoptosis [33]. In this study, the MCF-7 cells treated with evodiamine at earlier time point (compared to the proliferation assay condition in **Figure 1**) showed a significant up-regulation in the expression of Nbk and Bax (**Figure 7** **and** **8**) and leaded to the activation of caspase and apoptosis. On the other hand, the expressions of ERα and ERβ were also down-regulated after treatment of evodiamine in MCF-7 cells. (**Figure 6**) This effect also decreased the proliferation of estrogen-dependent MCF-7 cells.

In conclusion, the effects of evodiamine include the decrease of cell proliferation and up-regulation of apoptosis-related molecules, such as caspase 7, PARP, Nbk, and Bax in MCF-7 cells. These results suggest that evodiamine may in part mediate through ER-inhibitory pathway to reduce breast cancer cell proliferation.
